# Targeted multiparametric magnetic resonance imaging/transrectal ultrasound‐guided (mpMRI/TRUS) fusion prostate biopsy versus systematic random prostate biopsy: A comparative real‐life study

**DOI:** 10.1002/cnr2.1962

**Published:** 2024-01-12

**Authors:** Trang H. N. Pham, Maximilian F. Schulze‐Hagen, Mohammad S. Rahnama'i

**Affiliations:** ^1^ Department of Urology Uniklinik Rheinisch‐Westfälische Technische Hochschule (RWTH) Aachen Aachen Germany; ^2^ Department of Radiology Städtisches Klinikum Solingen Solingen Germany; ^3^ Department of Urology Nij Smellinghe Hospital Drachten The Netherlands; ^4^ Society of Urological Research and Education (SURE) Heerlen The Netherlands

**Keywords:** fusion targeted biopsy, multiparametric magnetic resonance imaging, prostate cancer, radical prostatectomy, systematic random biopsy

## Abstract

**Background:**

Patients with suspected prostate cancer usually undergo transrectal ultrasound‐guided (TRUS) systematic biopsy, which can miss relevant prostate cancers and lead to overtreatment.

**Aims:**

The aim of this study was to evaluate the detection rate for prostate cancer in MR‐guided targeted biopsy (TB) and systematic biopsy (SB) in comparison with mpMRI of the prostate.

**Methods and results:**

Three hundred and eight men who underwent mpMRI due to elevated PSA values between 2015 and 2020 were studied at university hospital Aachen, Germany. MRI‐images were divided into cohorts with suspicious findings (PI‐RADS ≥ 3) and negative findings (PI‐RADS < 3). In patients with PI‐RADS ≥ 3 TB combined with SB was performed. A part of this group underwent RP subsequently. In patients with PI‐RADS < 3 and clinical suspicion SB was performed. In the PI‐RADS ≥ 3 group (*n* = 197), TB combined with SB was performed in 194 cases. Three cases were lost to follow‐up. Biopsy yielded 143 positive biopsies and 51 cases without carcinoma. TB detected 71% (102/143) and SB 98% (140/143) of the overall 143 carcinoma. Overall, 102 carcinomas were detected by TB, hereof 66% (67/102) clinically significant (Gleason ≥ 3+4) and 34% (35/102) clinically insignificant carcinoma (Gleason 3+3). SB detected 140 carcinomas, hereof 64% (90/140) csPCA and 36% (50/140) nsPCA. Forty‐one of the overall 143 detected carcinoma were only found by SB, hereof 46% (19/41) csPCA and 54% (22/41) nsPCA. Tumor locations overlapped in 44% (63/143) between TB and SB. In 25% (36/143), SB detected additional tumor foci outside the target lesions. 70/143 patients subsequently underwent RP. The detection of tumor foci was congruent between mpMRI and prostatectomy specimen in 79% (55/70) of cases. Tumor foci were mpMRI occult in 21% (15/70) of cases. In the group with negative mpMRI (*n* = 111), biopsy was performed in 81 cases. Gleason ≥ 3+4 carcinoma was detected in 7% and Gleason 3+3 in 24% cases.

**Conclusion:**

There was a notable number of cases in which SB detected tumor foci that were mpMRI occult and could have been missed by TB alone. Therefore, additional systematic random biopsy is still required. A supplemental random biopsy should be considered depending on the overall clinical suspicion in negative mpMRI.

## 1 INTRODUCTION

Transrectal ultrasound‐guided systematic prostate biopsy (TRUS‐SB) is used to diagnose prostate cancer (PCa) as proposed by Ouzzane et al.,^1^, ^2^ as ultrasound sensitivity to detect PCa is low. Currently, SB is offered to men with clinical suspicion, including raised prostate specific antigen (PSA) or a suspicious digital rectal exam.^3^

The Gleason score (GS) associated to PCa progression and management of the disease, is used to assess PCa. Because of sample inconsistencies, pre‐therapeutic risk evaluations based on SB may be unreliable.

As a consequence, clinically significant prostate cancers (csPCa) might be missed by this approach which can potentially result in undertreatment.^4^ On the other hand, overdiagnosis of clinically insignificant PCa might lead to overtreatment.^4^ TRUS‐biopsy also carries significant morbidity and can cause complications such as fever, rectal bleeding, hematuria, acute urinary retention, and sepsis.^5^

Multiparametric Magnetic Resonance Imaging (mpMRI) has proved to be a valuable screening tool for men having a clinical suspicion of PCa.^6^ MpMRI results coupled with ultrasound‐guided biopsy offer a higher diagnostic performance and can identify the site of csPCa more accurately.^7^

TB has been proven to be superior to SB in several trials.^8^ The PRECISION study reported that the approach of obtaining cores with TB alone showed a higher detection rate of csPCa and a lower detection rate of insignificant carcinomas.^6^ These study results influenced the recommendations of the European Association of Urology guidelines that recommend the performance of concomitant SB in order to minimize the possibility of missing targeted regions of interest and/or misleading mpMRI findings.^3^ This recommendation is based on the study findings which have shown that combining SB and TB improves the detection rates of csPCa.^9^

Whether an unremarkable mpMRI should make SB unnecessary and whether TB alone is sufficient to detect csPCa is currently under debate.

In our study, we aimed to compare mpMRI/TRUS fusion targeted biopsy with TRUS systematic random biopsy. The study is unique, as we have used the RP histology in our daily practice and compared the results of mpMRI with the whole mount histopathology after RP.

## 2 MATERIALS AND METHODS

Three hundred and eight patients who underwent a pre‐biopsy mpMRI of the prostate between 2015 and 2020 at the university hospital RWTH Aachen, Germany were included in this retrospective study. The study was conducted in compliance with the recommendations of the local ethics committee (EK 225/22).

Patient data such as age, PSA, digital rectal exam, prostate volume and prior biopsies were recorded. Clinical suspicion of PCa based on elevated PSA or abnormal digital rectal exam was used to indicate the use of mpMRI. TB combined with SB was recommended for patients with mpMRI results of PI‐RADS ≥ 3. Eventually, all patients who subsequently had a RP, were included in the cohort.

All included patients underwent mpMRI using a 3.0‐T MRI system with a multi‐channel surface coil. The scan protocol adhered to the recommendations for image acquisition according to PIRADS with T2‐weighted imaging in three planes, diffusion‐weighted imaging, and dynamic contrast‐enhanced imaging. All mpMRI images were reported independently and reviewed centralized by experienced radiologists according to the Prostate Imaging Reporting and Data System (PI‐RADS) v2.1 guidelines and assigned a PI‐RADS score.^3^, ^10^

Patients were divided into two groups according to the reports:

Group 1 included patients with suspicious mpMRI outcome (PI‐RADS ≥ 3).
Group 2 included patients with negative mpMRI outcome (PI‐RADS < 3).

For the assessment of sensitivity, specificity and positive and negative predictive value (PPV and NPV) of TB for the diagnosis of csPCa the PI‐RADS scores with cutoff‐values of PI‐RADS 3 and 4 were used, respectively.

Biopsy procedure in study group 1 was as follows: Patients with lesions identified on mpMRI with PI‐RADS ≥ 3 subsequently proceeded to a combined biopsy procedure, including a systematic 12‐core biopsy and an mpMRI/TRUS fusion targeted biopsy (*n* = 194). With mpMRI images superimposed on a TRUS image, TB was conducted on previously suspected MRI lesions. For each lesion, at least two cores were obtained.^11^ Fusion software technology was used for all TB. In addition, SB was performed without considering the location of the MRI lesion. Typically based on the European Association of Urology (EAU) guidelines, 12 cores were collected in an extended sextant template from lateral to medial of base, mid, and apex portions of the prostate on both sides.^3^

In a subgroup of 70 men, RP was conducted after biopsy which is considered the gold standard for treating localized prostate cancer as it samples the entire prostate. Multiple experienced pathologists assigned biopsy cores and prostatectomy whole‐mount pathology with GS. We defined a csPCa as GS ≥ 3+4 and/or any cancer involvement ≥50% in any biopsy core and clinically insignificant carcinomas as GS 3+3.^12^, ^13^

Biopsy procedure in study group 2 was as follows: Patients who had a clearly positive digital rectal exam or an inexplicable persistent elevation of PSA (*n* = 81) were subjected to a SB despite a negative mpMRI. The biopsy samples were reported and reviewed centralized by multiple experienced pathologists and assigned with GS.

### 2.1 Statistical analysis

Patient demographic, clinical parameters, digital rectal exam findings, and PSA values were collected from the medical record. All of the prior data and histology reports were entered into an Excel database. Descriptive statistics were presented by number (percentage) or median (interquartile range). Pearson's Chi‐square test was used to calculate the relationship between PI‐RADS score at mpMRI and GS at biopsy. Relation between variables were considered significant for *p* < .05.

## 3 RESULTS

**FIGURE 1:**
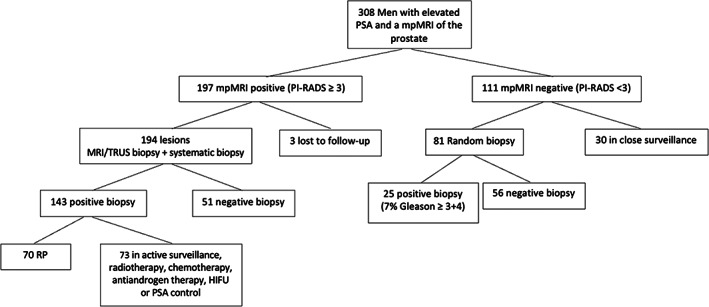
Flowchart. mpMRI, Multiparametric Magnetic Resonance Imaging; RP, radical prostatectomy.

A total of 308 patients underwent mpMRI which were analyzed and based on PI‐RADS classification divided into two groups. Group 1 (*n* = 197) had a positive (PI‐RADS ≥ 3) mpMRI outcome and group 2 (*n* = 111) had a negative (PI‐RADS < 3) mpMRI outcome (Figure 1).

The characteristics of the 308 patients are summarized in Table 1. The median age was 68 years (IQR 52–89 years), the median PSA level was 7.5 ng/mL (IQR 0.74–162 ng/mL), and the median prostate volume was 48 mL (IQR 14–215 mL). A total of 234 patients underwent previous biopsy.

**TABLE 1:** Patient characteristics.

Measure	Number of patients (%)	Median (IQR)
Age, years		68 (52–89)
PSA, ng/mL		7.5 (0.74–162)
Prostate volume, mL		48 (14–215)
Positive DRE	26 (8.4)	
PI‐RADS > 3 on MRI	197 (64)	
First biopsy	74 (24)	
Re‐biopsy	234 (76)	
Negative prior biopsy	108	
Positive prior biopsy	126	

Abbreviations: DRE, digital rectal exam; IQR, interquartile range; MRI, magnetic resonance imaging; PSA, prostate specific antigen.

**FIGURE 2:**
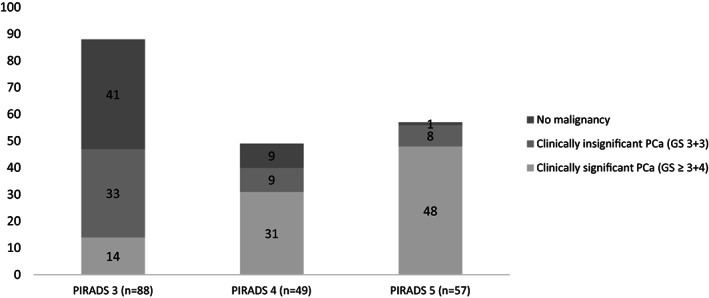
Correlation between PI‐RADS score and biopsy GS.

In group 1, TB combined with SB was performed in 194 cases, subsequently. Three cases did not receive any biopsy and were lost to follow‐up. The biopsy yielded 143 cases with a positive result and 51 cases were without evidence of PCa. The evaluation of the correlation between the relevant PI‐RADS lesions and the evidence of malignancy in biopsy showed that PI‐RADS score 3 was associated with csPCa (GS ≥ 3+4) at biopsy in 15.9% (14/88) of cases. While PI‐RADS score 4 was associated at biopsy with csPCa in 63.3% (31/49) of cases, and PI‐RADS score 5 was associated at biopsy with csPCa in 84.2% (48/57) of cases (Figure 2). The relation between these variables was significant (*χ* ^2^ (4, *N* = 194) = 89.6, *p* < .05).

The 51 patients in group 1 with a negative biopsy remained under PSA monitoring.

TB revealed 71.3% (102/143) of carcinomas, of which 2.1% (3/143) were identified by TB alone. However, TB missed 28.7% (41/143) of the PCa lesions. By contrast, SB revealed 97.9% (140/143) of carcinomas, of which 28.7% (41/143) were identified by SB alone. SB missed 2.1% (3/143) of the PCa lesions. Of those 102 carcinomas revealed by TB, clinically significant prostate cancer (csPCa) was detected in 65.7% (67/102), as compared with 64.3% (90/140) in the group with csPCa revealed by SB.

**FIGURE 3:**
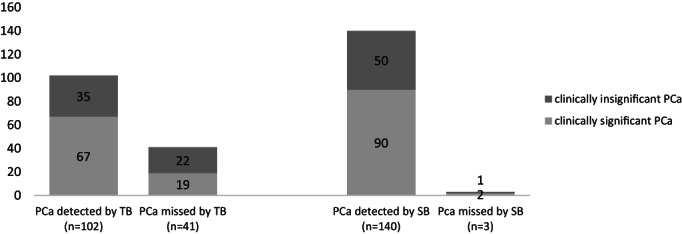
Detection rate of prostate cancer from targeted biopsy (TB) versus systematic biopsy (SB).

In this regard, SB had a higher rate in overall PCa detection in comparison to TB (97.9% vs. 71.3%). However, SB and TB did not differ in detection of csPCa significantly (64.3% vs. 65.7%) (Figure 3). The evaluation of the positive findings with respect to the GS is shown in Table 2.

**TABLE 2:** Gleason score distribution in systematic (SB) and targeted biopsy (TB).

Proportion of positive cores (%)	Gleason score					Total
	6	7	8	9	10	
In SB	50 (35.7)	50 (35.7)	19 (13.6)	18 (12.9)	3 (2.1)	140
In TB	35 (34.3)	29 (28.4)	15 (14.7)	19 (18.6)	4 (3.9)	102

Tables 3 and 4 present the diagnostic results for sensitivity, specificity, positive predictive value and negative predictive value for TB in mpMRI with PI‐RADS ≥ 3 against PI‐RADS ≥ 4.

**TABLE 3:** Contingency table for targeted biopsy (TB) to detect clinically significant prostate cancer (GS ≥ 3+4) in mpMRI with PI‐RADS ≥ 3.

	TB positive	TB negative	Total
csPCa	71	19	90
No cancer or cisPCa	31	73	104
Total	102	92	194

Abbreviations: cisPCa, clinically insignificant prostate cancer; csPCa, clinically significant prostate cancer.

**TABLE 4:** Contingency table for targeted biopsy (TB) to detect clinically significant prostate cancer (GS ≥ 3+4) in mpMRI with PI‐RADS ≥ 4.

	TB positive	TB negative	Total
csPCa	68	8	76
No cancer or cisPCa	15	15	30
Total	83	23	106

Abbreviations: cisPCa, clinically insignificant prostate cancer; csPCa, clinically significant prostate cancer.

In PI‐RADS ≥ 3 images, sensitivity of TB for csPCa was 79% and the negative predictive value was 79%. Specificity of TB was 70% and the probability of having a csPCa was 70% (PPV) (Table 3).

In PI‐RADS ≥ 4 images, sensitivity of TB for csPCa was 89% and negative predictive value was 65%. Specificity of TB was 50% with a positive predictive value of 82% (Table 4).

The laterality comparison of the lesions at TB for left, right and bilateral side, with the SB histologic report was concordant in 14.7%, 14.0%, and 15.4% of biopsies, respectively (Table 5).

**TABLE 5:** Prostate cancer localization comparison in biopsy cores.

Target side of PCa		Total (%)
In TB	In SB	
Left	Left	21 (14.7)^a^
Right	Right	20 (14)^a^
Bilateral	Bilateral	22 (15.4)^a^
Left *or* right	Bilateral	36 (25.2)
Left *or* right *or* bilateral	Negative	3 (2.1)
Negative	Left *or* right *or* bilateral	41 (28.7)
Total		143 (100)

^a^ Tumor location overlapping between TB and SB (44.1).

In 25.2% (36/143) of cases, SB detected additional lesions outside of the MRI‐targeted lesion side, for example, TB could reveal PCa on the left side of the prostate, but SB detected PCa on the left but also additionally on the right side.

In 28.7% (41/143) of cases only SB could reveal carcinomas, which were missed by TB.

In the subgroup of our study, we included men who had a positive biopsy and proceeded to RP after biopsy (70 of 143 patients). We compared the mpMRI findings with the whole‐mount section after RP. The laterality concordance of the lesions at mpMRI with the histologic report of the specimen after RP was confirmed in 24.3%, 21.4%, and 30% of cases for the right, left and bilateral side, respectively. A total of 75.7% (53/70) of the cases had matching sides at mpMRI and RP (Table 6). However, in 21.4% (15/70) of the cases tumor foci were mpMRI‐occult and were overseen by mpMRI.

**TABLE 6:** Concordance between target side at mpMRI and side of the RP specimen.

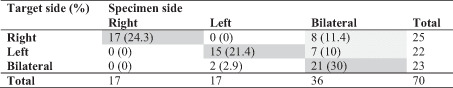

*Note*: Marked, absolute concordance between mpMRI and RP; hatched, mpMRI‐occult lesions.

The detection rate of prostate cancer lesions in mpMRI was 78.6% (55/70) that was affirmed in the histopathology of RP specimen.

The GS concordance rate between TB and combined biopsy (TB+SB) with final RP histology was 52.9% (37/70) and 64.3% (45/70), respectively. The addition of SB increased the GS concordance between biopsy and RP histology in this study. In the PI‐RADS 3 lesions, TB alone detected 31% (5/16) of csPCa (GS ≥ 3+4) and the combined biopsy (TB+SB) could detect 44% (7/16) of csPCa.

Correlation between PI‐RADS and definitive histologic report of the RP specimen are reported in the following table (Table 7). Regarding PI‐RADS 3 score, 75% of cases were associated with csPCa at definitive histologic report. PI‐RADS 4 score was associated with csPCa in 90.5% of cases and PI‐RADS 5 score in 97% of cases. Overall, a positive mpMRI was associated with csPCa at RP histologic report in 90% of cases (*χ* ^2^ (2, *N* = 70) = 5.79, *p* < .1).

**TABLE 7:** Correlation between PI‐RADS score and Gleason score (GS) of the RP specimen.

PI‐RADS (%)	Specimen report (GS)					Total
	6	7	8	9	10	
3	4 (25)	12 (75)	0 (0)	0 (0)	0 (0)	16
4	2 (9.5)	14 (66.7)	3 (14.3)	2 (9.5)	0 (0)	21
5	1 (3)	24 (72.7)	2 (6.1)	6 (18.2)	0 (0)	33

The rest of the men who did not undergo RP received different forms of further treatment such as radiotherapy, chemotherapy, antiandrogen therapy, high intensity focused ultrasound therapy, active surveillance or PSA monitoring.

In study group 2, the results were as follows: from the cohort of 308 men, 111 patients had a negative mpMRI outcome. Of them, in 81 men SB was performed despite negative mpMRI. Thirty men remained under PSA monitoring. In 30.9% (25/81) of the cases PCa was found. Clinically significant prostate cancer (GS ≥ 3+4) was detected in 7.4% (6/81) of cases and GS 3+3 carcinoma in 23.5% (19/81) of cases.

## 4 DISCUSSION

In this retrospective study, the detection rate for prostate cancer in TB and SB in comparison with mpMRI of the prostate was evaluated. In addition, histological reports of prostatectomy specimen were compared to the results of mpMRI, and test quality criteria for the detection of prostate carcinoma by TB in the presence of positive mpMRI findings were investigated. Moreover, the detection rates for PCa in the presence of negative mpMRI findings were analyzed.

In our study, the cancer detection rate in SB was substantially higher than in TB (97.9% vs. 71.3%). However, there was similar agreement in detecting any grade of csPCa (64.3% vs. 65.7%). This could partly be explained by the tumor size. Small tumors (<0.5 mL) could be easier randomly detected by SB. In line with our findings, Febres‐Aldana et al.^14^ showed that the PCa detection rate was higher in SB than in TB (51.8% vs. 44.6%).

The gold standard for reliable evidence of PCa localization and focality is the pathological report after RP. A study of Kam et al.,^15^ evaluating the accuracy of mpMRI and RP in carcinoma detection, found a 75% overall concordance of target lesion sites between the mpMRI scan and the RP histologic report in 235 patients, which is similar to our findings (75.7%). Our data are consistent with the previously reported studies with an overall concordance rate of 75.7% between mpMRI result and RP specimen report, and a slightly higher detection rate of csPCa with 75%, 90.5%, and 97% in PI‐RADS categories 3, 4, and 5, respectively. But we should note that using whole mount RP comes with a bias of selecting for those who underwent treatment presumably due to some high‐risk feature. These individuals may be more likely to harbor occult malignancy.

Our study found a slightly improved level of GS concordance between biopsy and RP histology with the inclusion of SB. Similarly, Kam et al.^16^ reported that GS was concordant between TB and combined biopsy with RP histologic report in 42% and 58% of 121 patients, respectively. Our data revealed that a mpMRI scan has a great accuracy and a high detection rate for csPCa with ascending PI‐RADS. According to this, targeted biopsy could show a high detection rate for csPCa (65.7%) and a large proportion of carcinomas (71.3%) could be identified by TB alone.

The incorporation of the PI‐RADS score as a pivotal element of the diagnostic imaging is a point of interest, especially in light of studies indicating the efficacy of TB without PI‐RADS.^17^ There are valid reasons for the continued use and incorporation of PI‐RADS in diagnostic imaging. It provides standardization, risk stratification, and facilitates communication and patient counseling. Additionally, its integration with other clinical data and its role in research contribute to its ongoing relevance in clinical practice.

The advantages of TB should be emphasized. With the accuracy of TB fewer GS 3+3 carcinomas could be detected, and fewer cores were taken. On the one hand, this reduces the incidence of complications associated with biopsy, especially in patients with a previous negative biopsy. On the other hand, it also minimizes the risk of overdiagnosis of clinically insignificant PCa and consequently overtreatment. Of course, it is important to consider that TB also missed a small proportion of csPCa (7.4%), which could result in undertreatment.

The problem with SB is that it also detects numerous GS 3+3 carcinomas. Nevertheless, SB is currently not redundant, as mpMRI does not yet have a detection rate of 100% and TB detected only 71% of PCa, but missed 29%. This is also in line with the current recommendations of the urologic guidelines. However, in the hands of experienced practitioners, mpMRI can be very accurate, and therefore concomitant SB should be reconsidered in patients who have inconspicuous TB, and be discussed with the patient depending on the PI‐RADS grade in the mpMRI.

Our study found that in patients with a negative mpMRI result (PI‐RADS 1 or 2) and elevated PSA values, SB found in 30.9% of cases PCa despite inconspicuous images, among them 7.4% csPCa. This finding is in line with previous study outcomes of Filson et al.,^18^ where csPCa was found by SB in 16% of men with a negative mpMRI outcome.

The detection rate of csPCa was relatively low (7.4%). In contrast, a great proportion of GS 3+3 carcinomas (30.9%) was diagnosed by additional SB. This raises the question of whether SB outweighs the detection of those few undetected csPCa with the overdetection of numerous Gleason 3+3 carcinomas.

Therefore, it is important to critically question whether a biopsy adds value and to weigh the advantages against the disadvantages. In cases of inconspicuous clinical course, it might be better to inform the patients about the residual risk for csPCa and to strive for a strategic follow‐up.^8^ However, in cases of high clinical suspicion (e.g., elevated PSA values), a biopsy should be considered, as tumor foci could be MRI‐occult. In such cases, SB alone would be sufficient.

For PI‐RADS 3, there is a controversy in the urologic and radiologic guidelines.

The German urological S3 guidelines recommend a biopsy protocol that involves a combination of TB and concomitant SB.^19^ Studies evaluating the PCa detection rates of combining TB with SB showed that a combined approach has the most reliable significance when compared with the individual procedures.^9^, ^18^

On the other hand, the radiologists recommend a follow‐up in patients with PI‐RADS 3, and only in case of an increase of PSA a biopsy should be considered.^20^

Regarding the correlation between the PI‐RADS score and the Gleason score (cf. Table 6), among the PI‐RADS 3 findings 25% were insignificant GS 3+3 carcinomas, whereas among PI‐RADS 4 and 5 only 9.5% and 3%, were GS 3+3, respectively. On the other hand, our study evaluated a detection rate of csPCa in PI‐RADS 3 with 75%, but 90.5% in PI‐RADS 4 and even 97% in PI‐RADS 5. This raises the question whether this fact supports that PI‐RADS 3 lesions should be targeted for biopsy as well. Therefore, in terms of overdiagnosis due to detection of clinically non‐significant cancers and underdiagnosis due to false‐negative results, test‐quality criteria of mpMRI with cutoff‐values of PI‐RADS 3 and 4 were investigated.

Indeed, in comparison to PI‐RADS ≥ 3, the positive predictive value improved from 70% to 82% and sensitivity from 79% to 89% in PI‐RADS ≥ 4, which implies a higher probability of having a csPCa, but partially due to the low findings of insignificant carcinomas in PI‐RADS 4 and 5. This led to less likelihood of overdiagnosis due to detection of Gleason 3+3 carcinomas.

However, the decreased negative predictive value from 79% to 65% and specificity from 70% to 50% implied a higher likelihood of underdiagnosis due to false‐negative results, making a SB still reasonable.

In cases of PI‐RADS 3 lesions, SB showed a quite high detection rate of csPCa and TB alone may not be sufficient since the risk of undetected csPCa appears to be substantial. Accordingly, TB+SB would seem to be recommendable in this scenario. This is also in accordance with the current German urological S3 guidelines.^19^ But ultimately, it is important to discuss the added value of a concomitant SB with the patient according to the individual clinical condition to avoid overdiagnosis.

In the presence of a PIRADS 4 or 5 lesion, the results of our study confirmed that TB has a very high reliability to be considered as sufficient to reach a conclusive diagnosis of csPCa. The detection rates of mpMRI in PI‐RADS 4 and 5 are 90% and 97%, respectively, and by that extremely accurate. In PI‐RADS 4, the data showed a high sensitivity of 89% and a PPV of 82%. Thus, it could be argued whether supplementary SB should only be performed in cases with negative prior TB. This is a point for serious consideration, since an additional biopsy would increase the number of cores needed and the risk for complications and would not bring any added value for the therapeutic decision. Current studies like the meta‐analysis published in 2019 by Kasivisvanathan et al.^21^ investigated and supported the strategy where TB represented an alternative to SB, as the detection rate of PCa was exceptionally high.

The differences in detection rates may have different ramifications on clinical practices and outcomes for patients. Higher detection rates with TB may impact treatment decisions and risk stratification. For example, if TB yields higher detection rates, it may lead to more accurate staging and risk assessment, influencing the choice of treatment options, such as active surveillance, surgery, or radiation therapy. It may also affect the quality of life for patients. If TB results in more accurate diagnoses and appropriate treatment choices for individual patients. It can reduce the likelihood of unnecessary procedures or overtreatment, potentially improving patients' long‐term quality of life and compliance with follow‐up recommendations as well, as there might be less discomfort and invasiveness with TB than SB. Of course, there is also the economic ramifications. Higher detection rates with TB may lead to cost savings in the long run if it reduces the need for additional diagnostic tests or treatment adjustments.

The reliability and accuracy of the current 3T mpMRI have been brought into question concerning their ability to detect variant histology of PCa or aberrant growth patterns, such as the cribriform pattern.^22^, ^23^, ^24^ It is crucial to acknowledge the significance of these entities in prostate cancer and their potential impact on prognosis, akin to their recognized role in both prostate and bladder cancer. These nuanced histological features, significant for prognosis, may not be adequately captured by standard imaging techniques, possibly introducing systematic biases in diagnostic accuracy and subsequent treatment decisions based on imaging findings.

In our study, while we primarily focused on the transrectal approach due to its widespread adoption, it is crucial to acknowledge the emerging prominence of the transperineal approach. The transperineal method has garnered attention for its perceived advantages, heralded not only for its potential to mitigate infectious complications but also for its improved diagnostic precision. Moreover, its efficacy in the context of repeat biopsies presents a compelling argument for its consideration as the new standard of care (SOC).^25^, ^26^, ^27^

Our study has several limitations: first, our data are acquired and analyzed in a retrospective, single‐center study design that included patients who had biopsies in the urology department. Patients who had active surveillance or radiotherapy were excluded from the part analyzing RP data. Second, our sample size was small and included a cohort of 194 patients who had a mpMRI and a combined biopsy. A larger group of patients would have been preferable although comparable studies did not include higher sample numbers. There is also a chance of targeting inaccuracy in this study. Third, possible bias must be taken into consideration as the urologist who carried out the TB were not blinded and had seen the mpMRI images and the radiology report and hence knew about the possible suspicious areas which could have led automatically to a cognitive mpMRI/TRUS‐targeted fusion biopsy.

Forth, our study primarily centered on the transrectal approach, thus overlooking the growing significance and benefits associated with the transperineal technique, which may limit the generalizability of our findings in light of these emerging trends. Fifth, aberrant growth patterns such as cribriform pattern and intraductal carcinoma of the prostate have not been taken into consideration in our study. This absence is significant, as these patterns have been associated with prognosis in PCa and have implications for treatment decisions and accuracy of the current 3T mpMRI. The inability to identify these patterns in our dataset may limit the applicability of our results to cases with these patterns and lead to potential systematic biases. Still, the absence of these patterns may not necessarily reflect their true absence in the patient population and may be due to specific study constraints.

## 5 CONCLUSION

The results indicate that a SB should be considered in the presence of a negative mpMRI scan depending on the overall clinical suspicion, and a general weighing of the pros and cons of a supplemental SB with patients is important. The majority of carcinomas was detectable by TB alone. However, TB alone missed tumor foci that were mpMRI occult. The combination of SB and TB exhibits a higher overall detection rate for csPCa.

## AUTHOR CONTRIBUTIONS

**Trang H. N. Pham:** Conceptualization (lead); data curation (lead); formal analysis (lead); visualization (lead); writing – original draft (lead). **Maximilian F. Schulze‐Hagen:** Formal analysis (supporting); writing – review and editing (supporting). **Mohammad S. Rahnama'i:** Conceptualization (supporting); data curation (supporting); formal analysis (supporting); writing – review and editing (supporting).

## CONFLICT OF INTEREST STATEMENT

The authors have stated explicitly that there are no conflicts of interest in connection with this article.

### ETHICS STATEMENT

The study was conducted in compliance with the recommendations of the local ethics committee (EK 225/22). Consent to participate: The study was retrospective, so (in accordance with the ethical approval) no written informed consent was obtained.

## Data Availability

The raw data supporting the conclusions of this article will be made available by the authors, without undue reservation.
